# 1,7-Dimethyl­penta­cyclo­[5.4.0.0^2,6^.0^3,10^.0^5,9^]undecane-8,11-dione

**DOI:** 10.1107/S1600536810025055

**Published:** 2010-07-03

**Authors:** Sai Kumar Chakka, Oluseye K. Onajole, Thavendran Govender, Glenn E. M. Maguire, Hong Su, Hendrik G. Kruger

**Affiliations:** a School of Chemistry, University of KwaZulu-Natal, Durban 4000, South Africa; b School of Pharmacy and Pharmacology, University of KwaZulu-Natal, Durban 4000, South Africa; c School of Chemistry, University of Cape Town, South Africa

## Abstract

The structure of the title compound, C_13_H_14_O_2_, a penta­cyclo­undecane cage derivative, exhibits unusual C*sp* ^3^—C*sp* ^3^ single-bond lengths ranging from 1.505 (3) to 1.607 (2) Å and strained bond angles as small as 88.7 (1)° and as large as 121.0 (2)°. In this *meso* compound, an inter­nal non-crystallographic mirror plane exists, bis­ecting the mol­ecule. In the crystal, weak C—H⋯O hydrogen bonds link the mol­ecules into an infinite spiral about a twofold screw axis along the [100] direction.

## Related literature

For related literature and examples of PCU cage structures exhibiting C—C bond lengths that deviate from the norm, see: Flippen-Anderson *et al.* (1991 ▶); Bott *et al.* (1998 ▶); Linden *et al.* (2005 ▶); Kruger *et al.* (2006 ▶). For the crystal packing of analogous PCU cage structures, see: Kruger *et al.* (2006 ▶); Boyle *et al.* (2007*a* ▶,*b* ▶). For the synthesis, see: Mehta *et al.* (1981 ▶). For hydrogen bonding, see: Desiraju *et al.* (1999 ▶).
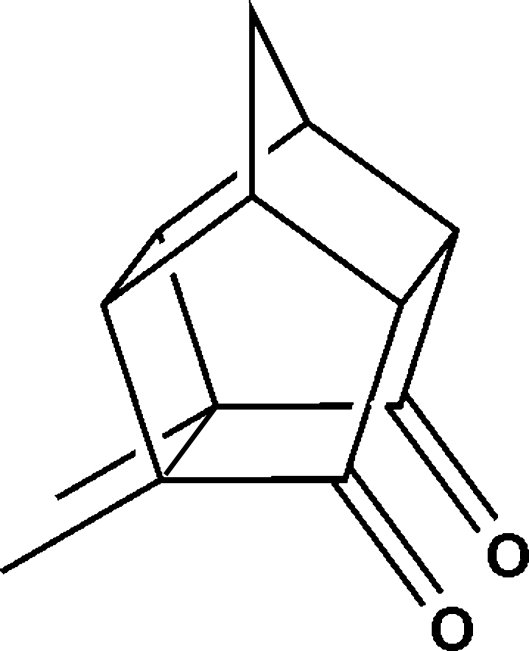


## Experimental

### 

#### Crystal data

C_13_H_14_O_2_
*M* *_r_* = 202.24
Orthorhombic, 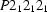

*a* = 7.7914 (2) Å
*b* = 8.2149 (3) Å
*c* = 15.4830 (5) Å
*V* = 991.00 (5) Å^3^
*Z* = 4
Cu *K*α radiation
μ = 0.72 mm^−1^
*T* = 173 K
0.32 × 0.25 × 0.21 mm

#### Data collection

Bruker Kappa DUO APEXII diffractometer
Absorption correction: multi-scan (*SADABS*; Sheldrick, 1997 ▶) *T*_min_ = 0.702, *T*_max_ = 0.753
4922 measured reflections
1055 independent reflections
1044 reflections with *I* > 2σ(*I*)
*R*_int_ = 0.020

#### Refinement

*R*[*F* ^2^ > 2σ(*F* ^2^)] = 0.033
*wR*(*F* ^2^) = 0.090
*S* = 1.06
1055 reflections
137 parameters
H-atom parameters constrained
Δρ_max_ = 0.21 e Å^−3^
Δρ_min_ = −0.18 e Å^−3^

### 

Data collection: *APEX2* (Bruker, 2006 ▶); cell refinement: *SAINT* (Bruker, 2006 ▶); data reduction: *SAINT*; program(s) used to solve structure: *SHELXS97* (Sheldrick, 2008 ▶); program(s) used to refine structure: *SHELXL97* (Sheldrick, 2008 ▶); molecular graphics: *ORTEP-3* (Farrugia, 1997 ▶); software used to prepare material for publication: *SHELXL97*.

## Supplementary Material

Crystal structure: contains datablocks I, global. DOI: 10.1107/S1600536810025055/hb5509sup1.cif

Structure factors: contains datablocks I. DOI: 10.1107/S1600536810025055/hb5509Isup2.hkl

Additional supplementary materials:  crystallographic information; 3D view; checkCIF report

## Figures and Tables

**Table 1:** Selected bond lengths (Å)

C1—C2	1.525 (3)
C1—C7	1.529 (2)
C2—C3	1.546 (2)
C2—C6	1.549 (2)
C3—C4	1.515 (3)
C3—C8	1.587 (2)
C4—C5	1.520 (2)
C5—C12	1.519 (2)
C5—C6	1.560 (2)
C5—C10	1.607 (2)
C6—C11	1.551 (3)
C7—C8	1.549 (2)
C7—C11	1.553 (3)
C8—C9	1.515 (3)
C9—C10	1.523 (3)
C10—C13	1.505 (3)
C10—C11	1.560 (2)

**Table 2:** Hydrogen-bond geometry (Å, °)

*D*—H⋯*A*	*D*—H	H⋯*A*	*D*⋯*A*	*D*—H⋯*A*
C2—H2⋯O2^i^	1.00	2.58	3.303 (2)	129
C3—H3⋯O2^ii^	1.00	2.59	3.335 (2)	131

Symmetry codes: (i) 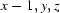; (ii) 
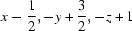
.

**Fig. 1.**
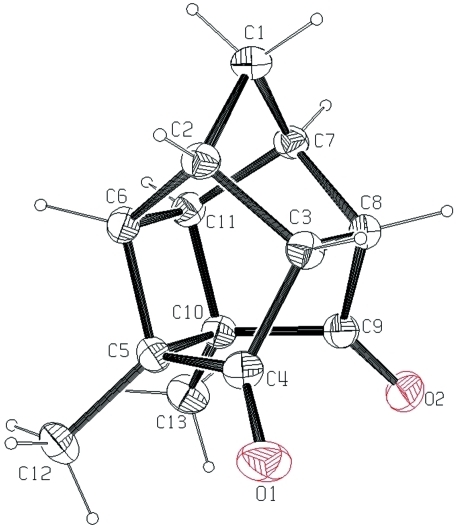
View of the molecular structure of (I) with non-H atoms drawn with 40% probability displacement ellipsoids and H atoms are shown as open circles.

**Fig. 2.**
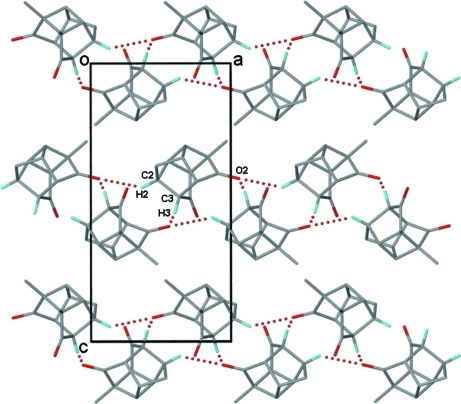
Projection viewed down the b axis of (I) showing the spirals up along the 2-fold screw axis in the [100] direction. Both the weak hydrogen bonds C3—H3···O2 and C2—H2···O2 are shown as dotted lines. All hydrogen atoms except H2 and H3 are omitted for clarity.

**Fig. 3.**
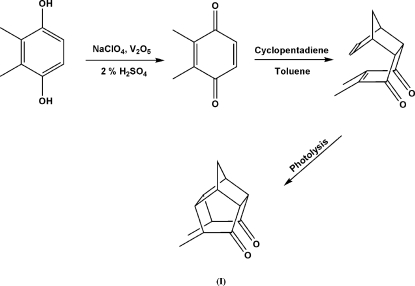
Preparation scheme for (I)

## supplementary crystallographic information

### Comment

As part of an ongoing study of the crystal structures and chemical reactivity of polycyclic pentacycloundecane (PCU) cage derivatives, the structure of the title compound, (I), was obtained (Scheme 1). Although the compound is known (Mehta *et al.*, 1981), its crystal structure has not been reported. Previous studies showed that PCU cage derivatives normally display C—C bond lengths which deviate from the expected value of 1.54 Å (see related literature). Similar phenomneon on the C—C bond lengths for this structure is observed, as the lengths of 17 C*sp*^3^—C*sp*^3^ single bonds range from 1.505 (3) Å to 1.607 (2) Å, with the bond between C10—C13 being the shortest, while that between C5—C10 is the longest (see Table 1). The labelling scheme and molecular structure is presented in Figure 1. The atoms C5, C6, C11 and C10 form a slightly irregular square with r.m.s. deviation of fitted atoms 0.0007 Å and is a very strained system. The tetrahedral bond angles around C10 are the most strained with the smallest angle of 88.7 (1)° (C5—C10—C11) and the biggest angle of 121.0 (2)° (C11—C10—C13), deviating from the ideal tetrahedral angle of 109.5°. Other selected carbon atoms, which define the cage conformation and which are coplanar with r.m.s. deviation of the fitted atoms smaller than 0.01 Å, are the following (with r.m.s. deviation of the fitted atoms in bracket): C10, C5, C4 and C9 (0.0034 Å); C4, C9, C8 and C3 (0.0010 Å); C3, C8, C7 and C2 (0.0019 Å); C2, C7, C11 and C6 (0.0004 Å). In the molecule of this *meso* compound an internal mirror plane exists, bisecting C1 and the middle points of bonds C8—C3, C11—C6 and C10—C5. We noted a number of weak hydrogen bonds of the type C—H···O=C presented in this structure (Desiraju *et al.*, 1999) (see Table 2). The molecules form a infinite right-hand spiral about a two fold screw-axis along the [100] direction *via* hydrogen bond C3—H3···O2 (see Figure 2).

### Experimental

In a 250 ml round-bottomed flask covered with tin foil was placed 2,3-dimethyl hydroquinone (4.00 g, 0.03 mmol), sodium chlorate (1.73 g, 0.01 mmol), 2% H_2_SO_4_ (36 ml) and 50 mg of vanadium pentoxide (catalyst). The mixture was stirred overnight, and the product, 2,3-dimethylbenzoquinone, was extracted with dichloromethane, dried over sodium sulfate, filtered and the filterate concentrated *in vacuo* to obtain 3.00 g (76%). To a vigorously stirring solution of the dried product (3.00 g, 0.02 mmol) in toluene (12 ml) cooled to 273 K, freshly cracked cyclopentadiene (1.67 g, 0.025 mmol) was added. The mixture was kept at 273 K for 4 h, after which the solution was allowed to attain ambient temperature over night. The solution was poured into an evaporating dish and placed in a fumehood to evaporate the toluene, yielding the adduct as a crude brown oil (4.20 g). Without further purification, the material was dissolved in ethyl acetate and exposed to sunlight until a clear solution was obtained (two weeks). The solvent was removed *in vacuo* to obtain a crude product, which was purified on silica gel, using a mobile phase of 6:4 hexane/ethyl acetate. The title compound was obtained as a pure white crystalline solid (3.20 g, 72%), mp 381–382 K. ^1^H NMR [CDCl3, 400 MHz]: δ = 0.99 (s, 6 H, CH3), 1.85 (d, 1 H, J= 11.2 Hz, CH2), 1.99 (d, 1 H, J= 11.1 Hz, CH2), 2.68 (s, 2 H, CH), 2.73 (s, 2 H, CH), 2.81 (s, 2 H, CH). ^13^C NMR [CDCl3, 100 MHz]: δ = 11.4 (*q*), 41.1 (*t*), 43.3 (*d*), 44.2 (*d*), 54.7 (*d*), 213.8 (*s*). IR (ATR): 2958, 1741, 1453,1282, 1073, 1023, 903, 869, 660 and 457 cm^-1^. Colourless prisms of (I) were grown by slow evaporation of a solution of the title compound in methanol, at ambient temperature.  The synthesis is summarised in Fig. 3.

### Refinement

The locations of the hydrogen atoms were found in a difference map and then positioned geometrically and allowed to ride on their respective parent atoms, with C—H bond lengths of 1.00 (CH), 0.99 (CH_2_), or 0.98 (CH_3_). They were then refined with a riding model with *U*_iso_(H) = 1.5Ueq(CH_3_) and *U*_iso_(H) = 1.2Ueq(*X*) for *X* = CH or CH_2_. When the data were unmerged, the Flack absolute structure parameter refined to -0.07 with s.u. 0.25.   Because of the large s.u., in the final refinement, the Friedel pairs were merged.

### Crystal data

**Table:**

C_13_H_14_O_2_	*D*_x_ = 1.356 Mg m^−^^3^
*M**_r_* = 202.24	Melting point: 382 K
Orthorhombic, *P*2_1_2_1_2_1_	Cu *K*α radiation, λ = 1.54184 Å
Hall symbol: P 2ac 2ab	Cell parameters from 4922 reflections
*a* = 7.7914 (2) Å	θ = 5.7–68.4°
*b* = 8.2149 (3) Å	µ = 0.72 mm^−^^1^
*c* = 15.4830 (5) Å	*T* = 173 K
*V* = 991.00 (5) Å^3^	Prism, colourless
*Z* = 4	0.32 × 0.25 × 0.21 mm
*F*(000) = 432	

### Data collection

**Table:**

Bruker Kappa DUO APEXII diffractometer	1055 independent reflections
Radiation source: fine-focus sealed tube	1044 reflections with *I* > 2σ(*I*)
graphite	*R*_int_ = 0.020
0.5° φ scans and ω scans	θ_max_ = 68.4°, θ_min_ = 5.7°
Absorption correction: multi-scan (*SADABS*; Sheldrick, 1997)	*h* = −9→9
*T*_min_ = 0.702, *T*_max_ = 0.753	*k* = −9→9
4922 measured reflections	*l* = −18→13

### Refinement

**Table:**

Refinement on *F*^2^	Secondary atom site location: difference Fourier map
Least-squares matrix: full	Hydrogen site location: inferred from neighbouring sites
*R*[*F*^2^ > 2σ(*F*^2^)] = 0.033	H-atom parameters constrained
*wR*(*F*^2^) = 0.090	*w* = 1/[σ^2^(*F*_o_^2^) + (0.0627*P*)^2^ + 0.1901*P*] where *P* = (*F*_o_^2^ + 2*F*_c_^2^)/3
*S* = 1.06	(Δ/σ)_max_ < 0.001
1055 reflections	Δρ_max_ = 0.21 e Å^−^^3^
137 parameters	Δρ_min_ = −0.18 e Å^−^^3^
0 restraints	Extinction correction: *SHELXL97* (Sheldrick, 2008), Fc^*^=kFc[1+0.001xFc^2^λ^3^/sin(2θ)]^-1/4^
0 constraints	Extinction coefficient: 0.0178 (18)
Primary atom site location: structure-invariant direct methods	

### Special details

**Table:**

Experimental. Half sphere of data collected using *COLLECT* strategy (Nonius, 2000). Crystal to detector distance = 30 mm; combination of φ and ω scans of 0.5°, 40 s per °, 2 iterations.
Geometry. All e.s.d.'s (except the e.s.d. in the dihedral angle between two l.s. planes) are estimated using the full covariance matrix. The cell e.s.d.'s are taken into account individually in the estimation of e.s.d.'s in distances, angles and torsion angles; correlations between e.s.d.'s in cell parameters are only used when they are defined by crystal symmetry. An approximate (isotropic) treatment of cell e.s.d.'s is used for estimating e.s.d.'s involving l.s. planes.
Refinement. Refinement of *F*^2^ against ALL reflections. The weighted *R*-factor *wR* and goodness of fit *S* are based on *F*^2^, conventional *R*-factors *R* are based on *F*, with *F* set to zero for negative *F*^2^. The threshold expression of *F*^2^ > σ(*F*^2^) is used only for calculating *R*-factors(gt) *etc*. and is not relevant to the choice of reflections for refinement. *R*-factors based on *F*^2^ are statistically about twice as large as those based on *F*, and *R*- factors based on ALL data will be even larger.

### Fractional atomic coordinates and isotropic or equivalent isotropic displacement parameters (Å^2^)

**Table:**

	*x*	*y*	*z*	*U*_iso_*/*U*_eq_	
O1	0.7584 (2)	0.32372 (19)	0.54950 (9)	0.0395 (4)	
C1	0.4669 (2)	0.6969 (2)	0.36778 (12)	0.0255 (4)	
H1A	0.4395	0.7912	0.4051	0.031*	
H1B	0.3858	0.6915	0.3187	0.031*	
O2	1.05692 (19)	0.5921 (2)	0.41443 (11)	0.0381 (4)	
C2	0.4768 (2)	0.5374 (2)	0.41804 (11)	0.0233 (4)	
H2	0.3674	0.5019	0.4461	0.028*	
C3	0.6306 (2)	0.5612 (2)	0.47985 (11)	0.0232 (4)	
H3	0.5988	0.6088	0.5371	0.028*	
C4	0.7082 (2)	0.3924 (2)	0.48546 (12)	0.0251 (4)	
C5	0.7092 (2)	0.3279 (2)	0.39345 (12)	0.0229 (4)	
C6	0.5531 (2)	0.4167 (2)	0.35149 (12)	0.0231 (4)	
H6	0.4697	0.3474	0.3190	0.028*	
C7	0.6546 (2)	0.6948 (2)	0.33893 (11)	0.0224 (4)	
H7	0.6912	0.7889	0.3023	0.027*	
C8	0.7553 (2)	0.6726 (2)	0.42436 (12)	0.0229 (4)	
H8	0.7872	0.7772	0.4532	0.027*	
C9	0.9079 (2)	0.5714 (2)	0.39604 (12)	0.0248 (4)	
C10	0.8361 (2)	0.4397 (2)	0.33708 (11)	0.0231 (4)	
C11	0.6756 (2)	0.5243 (2)	0.29697 (11)	0.0230 (4)	
H11	0.6630	0.5181	0.2328	0.028*	
C12	0.7267 (3)	0.1443 (2)	0.38573 (13)	0.0316 (5)	
H12A	0.7260	0.1133	0.3246	0.047*	
H12B	0.6306	0.0915	0.4154	0.047*	
H12C	0.8350	0.1096	0.4122	0.047*	
C13	0.9672 (3)	0.3567 (3)	0.28080 (13)	0.0334 (5)	
H13A	0.9104	0.2740	0.2452	0.050*	
H13B	1.0541	0.3046	0.3172	0.050*	
H13C	1.0223	0.4374	0.2433	0.050*	

### Atomic displacement parameters (Å^2^)

**Table:**

	*U*^11^	*U*^22^	*U*^33^	*U*^12^	*U*^13^	*U*^23^
O1	0.0546 (10)	0.0372 (8)	0.0267 (7)	0.0111 (8)	−0.0061 (7)	0.0058 (6)
C1	0.0229 (9)	0.0265 (9)	0.0269 (9)	0.0046 (7)	−0.0001 (7)	0.0022 (8)
O2	0.0216 (7)	0.0364 (8)	0.0564 (10)	−0.0003 (6)	−0.0065 (7)	−0.0079 (7)
C2	0.0193 (8)	0.0247 (9)	0.0259 (8)	0.0007 (7)	0.0027 (7)	0.0016 (7)
C3	0.0255 (8)	0.0245 (9)	0.0195 (8)	0.0020 (8)	0.0024 (7)	−0.0010 (7)
C4	0.0250 (8)	0.0264 (9)	0.0240 (8)	0.0009 (8)	0.0010 (7)	0.0017 (7)
C5	0.0237 (9)	0.0195 (9)	0.0256 (9)	−0.0004 (7)	0.0012 (7)	−0.0011 (7)
C6	0.0218 (8)	0.0240 (9)	0.0235 (8)	−0.0016 (8)	−0.0017 (7)	−0.0019 (7)
C7	0.0231 (9)	0.0208 (8)	0.0232 (8)	−0.0001 (8)	−0.0001 (7)	0.0032 (7)
C8	0.0227 (8)	0.0201 (8)	0.0257 (9)	−0.0009 (8)	−0.0029 (8)	−0.0014 (7)
C9	0.0224 (9)	0.0241 (9)	0.0280 (9)	−0.0012 (8)	0.0004 (7)	0.0030 (7)
C10	0.0215 (9)	0.0240 (9)	0.0237 (8)	0.0016 (8)	0.0023 (7)	−0.0003 (7)
C11	0.0236 (9)	0.0255 (9)	0.0200 (8)	0.0001 (8)	−0.0001 (7)	−0.0013 (7)
C12	0.0421 (11)	0.0204 (9)	0.0324 (9)	0.0009 (9)	0.0015 (9)	−0.0011 (7)
C13	0.0315 (11)	0.0344 (11)	0.0344 (9)	0.0057 (9)	0.0092 (9)	−0.0020 (9)

### Geometric parameters (Å, °)

**Table:**

O1—C4	1.206 (2)	C6—H6	1.0000
C1—C2	1.525 (3)	C7—C8	1.549 (2)
C1—C7	1.529 (2)	C7—C11	1.553 (3)
C1—H1A	0.9900	C7—H7	1.0000
C1—H1B	0.9900	C8—C9	1.515 (3)
O2—C9	1.207 (3)	C8—H8	1.0000
C2—C3	1.546 (2)	C9—C10	1.523 (3)
C2—C6	1.549 (2)	C10—C13	1.505 (3)
C2—H2	1.0000	C10—C11	1.560 (2)
C3—C4	1.515 (3)	C11—H11	1.0000
C3—C8	1.587 (2)	C12—H12A	0.9800
C3—H3	1.0000	C12—H12B	0.9800
C4—C5	1.520 (2)	C12—H12C	0.9800
C5—C12	1.519 (2)	C13—H13A	0.9800
C5—C6	1.560 (2)	C13—H13B	0.9800
C5—C10	1.607 (2)	C13—H13C	0.9800
C6—C11	1.551 (3)		
			
C2—C1—C7	95.26 (14)	C1—C7—H7	115.5
C2—C1—H1A	112.7	C8—C7—H7	115.5
C7—C1—H1A	112.7	C11—C7—H7	115.5
C2—C1—H1B	112.7	C9—C8—C7	102.44 (14)
C7—C1—H1B	112.7	C9—C8—C3	108.72 (15)
H1A—C1—H1B	110.2	C7—C8—C3	102.71 (14)
C1—C2—C3	104.26 (15)	C9—C8—H8	113.9
C1—C2—C6	103.29 (14)	C7—C8—H8	113.9
C3—C2—C6	101.25 (14)	C3—C8—H8	113.9
C1—C2—H2	115.4	O2—C9—C8	127.53 (19)
C3—C2—H2	115.4	O2—C9—C10	126.49 (19)
C6—C2—H2	115.4	C8—C9—C10	105.96 (15)
C4—C3—C2	103.24 (14)	C13—C10—C9	114.82 (16)
C4—C3—C8	108.33 (14)	C13—C10—C11	121.02 (15)
C2—C3—C8	102.26 (13)	C9—C10—C11	102.51 (14)
C4—C3—H3	114.0	C13—C10—C5	118.23 (16)
C2—C3—H3	114.0	C9—C10—C5	107.86 (14)
C8—C3—H3	114.0	C11—C10—C5	88.69 (13)
O1—C4—C3	127.20 (18)	C6—C11—C7	102.81 (13)
O1—C4—C5	127.29 (18)	C6—C11—C10	91.31 (13)
C3—C4—C5	105.51 (15)	C7—C11—C10	108.66 (14)
C12—C5—C4	114.86 (16)	C6—C11—H11	116.8
C12—C5—C6	120.11 (16)	C7—C11—H11	116.8
C4—C5—C6	102.91 (15)	C10—C11—H11	116.8
C12—C5—C10	117.97 (16)	C5—C12—H12A	109.5
C4—C5—C10	108.24 (14)	C5—C12—H12B	109.5
C6—C5—C10	89.23 (13)	H12A—C12—H12B	109.5
C2—C6—C11	103.48 (14)	C5—C12—H12C	109.5
C2—C6—C5	108.76 (14)	H12A—C12—H12C	109.5
C11—C6—C5	90.77 (13)	H12B—C12—H12C	109.5
C2—C6—H6	116.8	C10—C13—H13A	109.5
C11—C6—H6	116.8	C10—C13—H13B	109.5
C5—C6—H6	116.8	H13A—C13—H13B	109.5
C1—C7—C8	103.67 (14)	C10—C13—H13C	109.5
C1—C7—C11	103.47 (15)	H13A—C13—H13C	109.5
C8—C7—C11	101.38 (14)	H13B—C13—H13C	109.5
			
C7—C1—C2—C3	52.94 (15)	C2—C3—C8—C7	−0.35 (17)
C7—C1—C2—C6	−52.54 (16)	C7—C8—C9—O2	134.3 (2)
C1—C2—C3—C4	−145.63 (14)	C3—C8—C9—O2	−117.5 (2)
C6—C2—C3—C4	−38.63 (17)	C7—C8—C9—C10	−44.16 (18)
C1—C2—C3—C8	−33.19 (16)	C3—C8—C9—C10	64.06 (18)
C6—C2—C3—C8	73.81 (15)	O2—C9—C10—C13	−15.9 (3)
C2—C3—C4—O1	−136.5 (2)	C8—C9—C10—C13	162.55 (16)
C8—C3—C4—O1	115.6 (2)	O2—C9—C10—C11	−149.1 (2)
C2—C3—C4—C5	43.41 (18)	C8—C9—C10—C11	29.38 (17)
C8—C3—C4—C5	−64.51 (17)	O2—C9—C10—C5	118.2 (2)
O1—C4—C5—C12	18.7 (3)	C8—C9—C10—C5	−63.30 (18)
C3—C4—C5—C12	−161.21 (16)	C12—C5—C10—C13	−0.9 (3)
O1—C4—C5—C6	151.1 (2)	C4—C5—C10—C13	131.62 (18)
C3—C4—C5—C6	−28.88 (18)	C6—C5—C10—C13	−125.04 (17)
O1—C4—C5—C10	−115.4 (2)	C12—C5—C10—C9	−133.26 (18)
C3—C4—C5—C10	64.62 (18)	C4—C5—C10—C9	−0.7 (2)
C1—C2—C6—C11	33.43 (17)	C6—C5—C10—C9	102.62 (16)
C3—C2—C6—C11	−74.33 (16)	C12—C5—C10—C11	124.02 (18)
C1—C2—C6—C5	128.94 (15)	C4—C5—C10—C11	−103.44 (15)
C3—C2—C6—C5	21.18 (17)	C6—C5—C10—C11	−0.10 (12)
C12—C5—C6—C2	133.27 (18)	C2—C6—C11—C7	−0.07 (17)
C4—C5—C6—C2	4.12 (18)	C5—C6—C11—C7	−109.58 (14)
C10—C5—C6—C2	−104.42 (15)	C2—C6—C11—C10	109.40 (14)
C12—C5—C6—C11	−122.20 (18)	C5—C6—C11—C10	−0.10 (12)
C4—C5—C6—C11	108.64 (14)	C1—C7—C11—C6	−33.26 (17)
C10—C5—C6—C11	0.10 (12)	C8—C7—C11—C6	73.97 (16)
C2—C1—C7—C8	−52.87 (16)	C1—C7—C11—C10	−129.09 (15)
C2—C1—C7—C11	52.62 (15)	C8—C7—C11—C10	−21.86 (17)
C1—C7—C8—C9	146.37 (15)	C13—C10—C11—C6	122.67 (18)
C11—C7—C8—C9	39.30 (17)	C9—C10—C11—C6	−107.91 (14)
C1—C7—C8—C3	33.62 (18)	C5—C10—C11—C6	0.10 (12)
C11—C7—C8—C3	−73.44 (16)	C13—C10—C11—C7	−133.34 (17)
C4—C3—C8—C9	0.2 (2)	C9—C10—C11—C7	−3.91 (17)
C2—C3—C8—C9	−108.38 (15)	C5—C10—C11—C7	104.10 (14)
C4—C3—C8—C7	108.24 (16)		

### Hydrogen-bond geometry (Å, °)

**Table:**

*D*—H···*A*	*D*—H	H···*A*	*D*···*A*	*D*—H···*A*
C2—H2···O2^i^	1.00	2.58	3.303 (2)	129
C3—H3···O2^ii^	1.00	2.59	3.335 (2)	131

Symmetry codes: (i) *x*−1, *y*, *z*; (ii) *x*−1/2, −*y*+3/2, −*z*+1.
